# Functional dynamics reveal the response of the crabapple (*Malus* sp.) phyllosphere microbiome to *Gymnosporangium yamadae* infection

**DOI:** 10.1128/msystems.00843-25

**Published:** 2025-09-15

**Authors:** Qianran Xu, Yonghua Zhang, Siqi Tao

**Affiliations:** 1 The Key Laboratory for Silviculture and Conservation of Ministry of Education, Beijing Forestry University 659924 , Beijing, People’s Republic of China; 2 Ecological Observation and Research Station of Heilongjiang Sanjiang Plain Wetlands, National Forestry and Grassland Administration 101622, Shuangyashan, People’s Republic of China; Boise State University, Boise, Idaho, USA

**Keywords:** metatranscriptome, phyllosphere microbiome, rust disease, *Malus *sp.

## Abstract

The phyllosphere microbiome plays a vital role in plant defense against airborne pathogens. In this study, we investigated microbial dynamics of crabapple (*Malus* ‘Kelsey’) phyllosphere across six stages of *Gymnosporangium yamadae* infection using metatranscriptomic sequencing. Infected leaves exhibited increased transcriptional alpha diversity of fungi, bacteria, and viruses, with a notable negative correlation between fungal and bacterial diversity. Microbial taxa in diseased leaves exhibited heightened transcriptional activity compared with healthy counterparts, with fungi progressively dominating the microbial community. Functional co-occurrence networks revealed increased complexity in infected leaves, suggesting a potential reorganization of the microbial interactions in response to pathogen invasion. These structural shifts were accompanied by significant functional changes, as revealed by functional annotation and random forest modeling, which showed significantly enhanced microbial carbohydrate metabolism and upregulated plant cell wall-degrading enzymes (e.g., PL3, CE8, and CE5) in diseased leaves. Key transcripts associated with these functions, originating from *Termitomyces* sp. J132 and *Alternaria alternata*, were opportunistically expressed and significantly positively correlated with host flavonoid glycosides, which serve as markers of rust lesion development. Additionally, diseased leaves showed activation of microbial pathways related to aromatic compound degradation and ascorbate metabolism, potentially contributing to redox homeostasis under host defense stress. At later infection stages, we detected high expression of genes encoding cell wall-degrading enzymes in *Saitozyma podzolica*. Together, our findings underscore the dynamic, functional role of the phyllosphere microbiome in modulating plant-pathogen interactions and shaping disease progression, providing a foundation for microbiome-informed strategies to enhance plant disease resistance.

**IMPORTANCE:**

Our study reveals stage-specific shifts in the transcriptional activity and functional capacity of the crabapple phyllosphere microbiome during *Gymnosporangium yamadae* infection. We identified key microbial taxa potentially involved in pathogen facilitation or antagonism and elucidated their roles in plant–pathogen interactions. These findings highlight the importance of the phyllosphere microbiome in regulating plant health and suggest new avenues for microbiome-based approaches to improving plant disease resilience.

## INTRODUCTION

The plant microbiome encompasses an entire community of microorganisms and their associated molecular spectrum within plant environments, forming a diverse and complex ecological network that significantly impacts plant health, resilience, and defense mechanisms (1–5). Current research underscores that plant fitness and immunity are not shaped solely by individual pathogens or beneficial species, but by the collective interactions within the microbiome (6).

Beneficial microbial communities, particularly those residing in the rhizosphere and phyllosphere, play critical roles in plant defense by modulating immune responses, competing with pathogens, and producing metabolites that enhance defense (3, 7–11). In the rhizosphere of tomato variety resistant to *Ralstonia solanacearum*, for instance, beneficial taxa such as *Flavobacteriaceae*, *Sphingomonadaceae,* and *Pseudomonadaceae* are more abundant than in the susceptible variety, providing a natural microbial buffer against pathogens (12). Similarly, *Arabidopsis thaliana* has been shown to recruit protective bacteria, such as *Microbacterium*, *Stenotrophomonas*, and *Xanthomonas*, which collectively induce systemic immunity against downy mildew (13). Such pathogen-induced enrichment of protective microbes aligns with the “cry for help” model, wherein stressed plants attract beneficial microbiota to reinforce defenses (13–15).

These insights highlight the potential of harnessing microbiome function to improve plant health (3). Functional metagenomics has uncovered gene patterns related to pathogenic responses, such as the enrichment of detoxification, biofilm formation, and chemotaxis genes during *Fusarium* wilt in peppers (16). Similarly, *Rhizoctonia solani* infection in sugar beets stimulates increases in *Chitinophagaceae* and *Flavobacteriaceae*, which produce antifungal enzymes and metabolites, adding a further layer of defense (17).

However, not all members of the plant microbiome are beneficial; some can be detrimental by forming harmful partnerships with pathogens, disrupting plant resilience, and facilitating disease progression (6, 18, 19). For example, *Verticillium dahliae* infection was shown to stabilize certain microbial networks within the bulk soil and the rhizosphere, with viruses becoming central players in the “pathobiome,” potentially aiding pathogen invasion and colonization (20). Similarly, studies on the diseases affecting tomato, tobacco, and other plants have shown that certain microbes associated with nematode pathogens release enzymes that degrade plant cell walls, which aids pathogen entry into roots and exacerbates disease progression (21, 22). This multifaceted relationship highlights that the plant microbiome’s role in pathogenesis is not a simple binary of pathogenic versus protective effects, but rather a context-dependent and dynamic interaction shaped by ecological and molecular factors (23).

Although research on plant–microbiome–pathogen interactions has traditionally focused on belowground compartments such as rhizosphere, the phyllosphere—the microbial community on the aboveground plant surfaces—is increasingly recognized for its crucial role in defending against foliar pathogens and supporting overall plant health (24–26). Emerging evidence further suggests the phyllosphere microbiome assembly is dynamic and context-dependent, with mechanisms varying in response to different pathogen pressures (27). For example, temporal analysis of the phyllosphere and rhizosphere microbiomes in soybean plants infected with *Phytophthora sojae* and *Septoria glycines* showed that the phyllosphere microbial communities exhibited more pronounced changes in structure and composition in response to pathogen invasion, including a marked increase in the abundance of saprophytic fungi (28). During disease progression, the phyllosphere microbial communities in apple, wheat, and tobacco were observed to exhibit higher diversity and more complex co-occurrence networks (8, 29–31). However, despite these insights into microbial community composition, most studies to date have primarily focused on cataloging microbial taxa associated with disease states. The functional responses of the phyllosphere microbiome—and their direct implications for plant health—remain largely underexplored (32).

Crabapple trees (*Malus* spp.), cherished in landscaping for their attractive shape and leaf color, face a significant threat from the rust fungus *Gymnosporangium yamadae*, which causes expanding brown lesions on leaves and markedly reduces their ornamental appeal (8, 33). The obligate biotrophic and unculturable nature of *G. yamadae* constrains conventional pathogen-host research, marking a microbiome-based approach essential (34). For example, cucumber leaves infected with powdery mildew showed increased bacterial alpha diversity and beneficial microbes with disease severity (35). In the context of *G. yamadae-Malus* interactions, a metatranscriptomic analysis of apple leaves (*M. domestica* cv. Fuji) compared fungal communities at the spermogonia (10 dpi, days post-inoculation) and aecia (30 dpi) stage, revealing significant shifts in community composition (30). Notably, the relative abundances of *Alternaria* and *Fonsecaea* increased during the later infection stage (30). Similarly, an amplicon-based study on apple (*M. domestica* cv. Gala) profiled endophytic microbiota at nine time points post-basidiospore inoculation, revealing a dynamic microbial trajectory characterized by an early increase and subsequent decline in diversity, coupled with opposing trends in community complexity and predicted functional profiles (36).

These findings point to temporally heterogeneous responses of the phyllosphere microbiome during the spermogonia and aecia stages, emphasizing the need for a more detailed, dynamic analysis of microbial functional shifts throughout the disease cycle. Building on this, a more recent time-series study examined fungal and bacterial communities, alongside key leaf metabolites, in two crabapple cultivars (‘Flame‘ and ‘Kelsey’) across six visually distinct stages of rust disease progression, from spermogonia formation to aecia maturation (8). The integrative study suggests that infected leaves may modulate disease progression by secreting specific metabolites—most notably flavonoids—that mediate the enrichment of potential beneficial microbes, consistent with the “cry for help” hypothesis (9). Flavonoids accumulate with lesion expansion and correlate with microbial shifts, indicating a dual role in symptom development and microbiome modulation (8, 37–39). Despite significant advances in characterizing the taxonomic diversity and structural dynamics of *Malus* phyllosphere microbiome under rust infection, our understanding of the functional contributions of these communities—and how they temporally coordinate with disease progression—remains limited. Gaining deeper insights into these functional dynamics is essential for elucidating how the phyllosphere microbiome adapts to *G. yamadae* invasion and how these changes impact host health.

This study aims to systematically investigate the functional response of the phyllosphere microbiome in crabapple leaves infected by *G. yamadae* across multiple stages of lesion expansion using metatranscriptomic technology. Specifically, our main objectives were to (i) characterize the diversity and composition of phyllosphere microbial transcriptomes at distinct stages of disease progression; (ii) identify differentially expressed microbial genes at each stage of infection and examine their dynamic expression profiles over time; (iii) elucidate the functional roles and activities of phyllosphere microbiota, and, in conjunction with host metabolic profiles from a previous study (8), and (iv) explore the relationships between key microbial functional genes and representative lesion-associated metabolites, particularly flavonoids, to assess their potential roles in disease development and host health.

## RESULTS

### Transcriptional alpha diversity and structure patterns in the phyllosphere during infection

**Fig 1:**
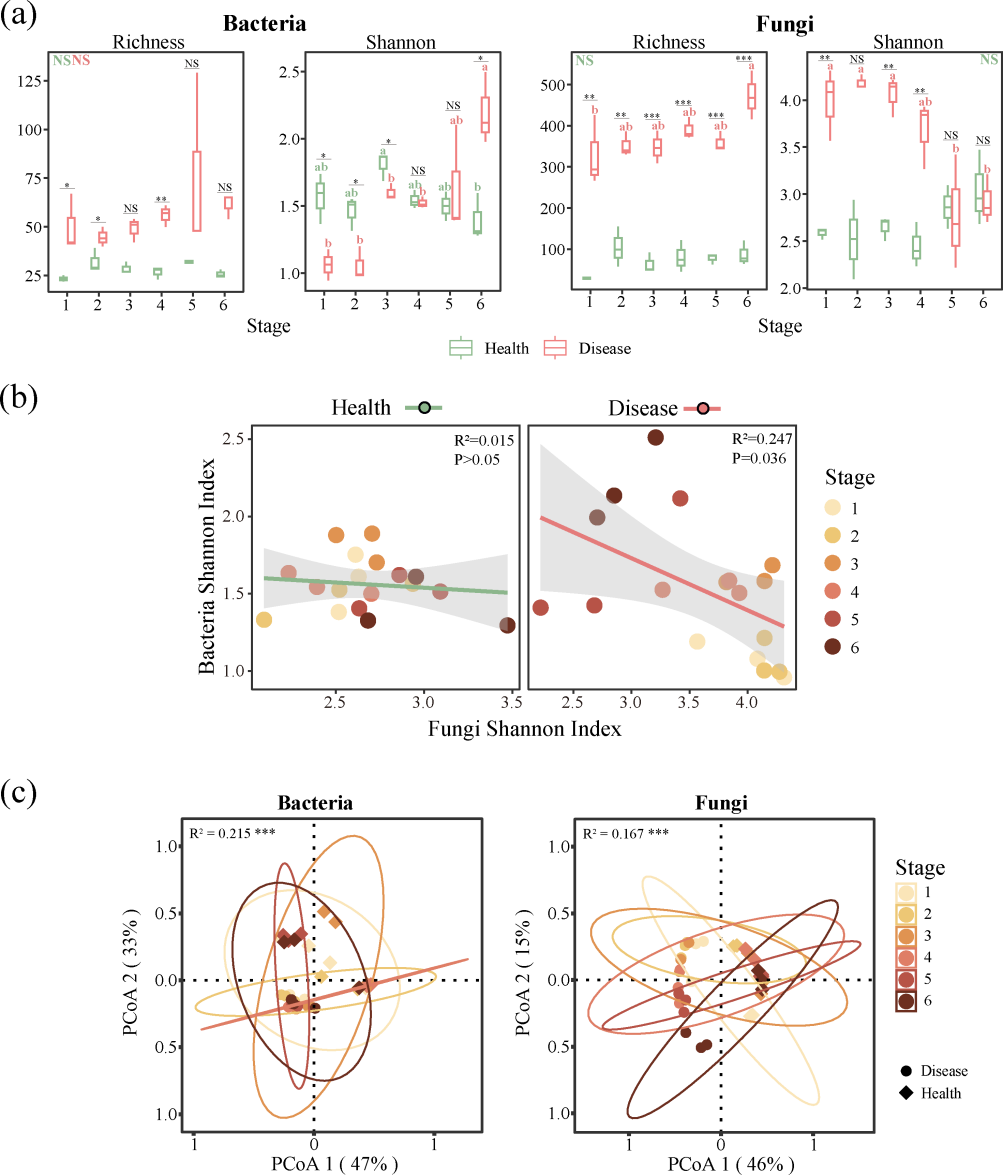
Phyllosphere transcriptomes, alpha diversity, and structural changes of bacteria and fungi under *Gymnosporangium yamadae* invasion. (**a**) The alpha diversity of bacterial and fungal transcriptomes in diseased leaves and healthy leaves across six developmental stages of crabapple rust disease. Box plots show the range of estimated values between the 25th and 75th percentiles, with the median, minimum, and maximum observed values within each data set. Different letters indicate statistically significant differences were determined using one-way ANOVA with Tukey-HSD *post hoc* test (*P* < 0.05) or Kruskal-Wallis with Wilcoxon’s test (*P* < 0.05). (**b**) The correlation of the Shannon index between bacterial and fungal transcriptomes. Solid lines represent the results of the simple linear regression models, while the surrounding gray band represents the 95% CI. (**c**) PCoA of bacterial and fungal transcriptomes based on the Bray-Curtis distance matrix, R², and *P* were calculated using the PERMANOVA test. Asterisks show the *P* significance level, *P < 0.05, **P < 0.01, ***P < 0.001, and ****P < 0.0001, and NS denotes no statistical significance.

The crabapple phyllosphere harbors a diverse microbial community whose composition and transcriptional activity shift markedly in response to *G. yamadae* infection (Fig. 1; Fig. S1; Table 1). Based on transcript expression levels and taxonomic annotations, microbial transcripts were classified into bacterial, fungal, viral, and archaeal categories. Species-level expression was quantified as the cumulative FPKM values for genes annotated to each species (Table S1). Archaea transcripts were excluded from subsequent diversity and structural analyses due to their extremely low abundance (Table 2; Table S1). Alpha diversity analysis revealed distinct trends in bacterial, fungal, and viral transcriptomes over the course of rust disease progression. Overall, fungal transcriptomes consistently exhibited higher alpha diversity than that of bacterial and viral transcriptomes (Fig. 1a; Fig. S1a). In bacterial transcriptomes, diseased leaves generally exhibited higher species richness compared with healthy leaves. The Shannon index in healthy leaves peaked at the 3rd stage and declined to its lowest value by the 6th stage. In early to mid-disease stages (Stages 1–4), healthy leaves displayed greater bacterial diversity at the transcriptome level than diseased leaves. However, as the lesions expanded, the Shannon index and Pielou’s evenness index of bacterial transcriptomes in diseased leaves incrementally increased, peaking at the sixth stage (Fig. 1a; Fig. S1b). For fungal transcriptomes, healthy leaves did not display any distinct patterns in diversity indices. However, *G. yamadae* infection initially resulted in a decline in transcript richness, which then rose to the peak at the final stage. Interestingly, the Shannon index, as well as Pielou’s evenness index, declined steadily in diseased leaves, an opposite trend compared to bacterial diversity (Fig. 1; Fig. S1b; Table S2). A simple linear regression analysis revealed a significant negative correlation between bacterial and fungal transcriptional Shannon indices, indicating that increased bacterial transcriptional diversity was associated with decreased fungal transcriptional diversity during disease progression (Fig. 1b). The viral alpha diversity did not exhibit any significant patterns across stages or between leaf conditions (Fig. S1a; Table S2).

**TABLE 1:** The effects of leaf condition and sampling stage on the structure of bacterial and fungal transcriptomes based on PERMANOVA with 999 permutations

Parameter	Bacteria			Fungi		
	*R*²	F	*P*	*R*²	F	*P*
Leaf condition (LC)	0.33476	27.4413	0.001	0.44416	50.7015	0.001
Sampling stage (SS)	0.15774	2.5861	0.004	0.17891	4.0845	0.001
Interaction (LC and SS)	0.21473	3.5204	0.001	0.16668	3.8054	0.001

**TABLE 2:** The relative expression abundances (%) of transcriptome components derived from unigenes across six disease stages in crabapple leaves

Domain	Stage 1		Stage 2		Stage 3		Stage 4		Stage 5		Stage 6	
	Disease	Health	Disease	Health	Disease	Health	Disease	Health	Disease	Health	Disease	Health
Bacteria	1.42%	0.66%	0.97%	0.55%	0.63%	0.68%	0.80%	0.44%	0.69%	1.02%	0.45%	1.03%
Fungi^*a*^	2.20%	0.03%	1.96%	0.36%	2.31%	0.11%	1.90%	0.25%	4.96%	22.19%	0.07%	0.10%
Viruses	0.46%	0.09%	0.37%	0.20%	0.37%	0.04%	0.52%	0.15%	0.04%	0.03%	0.44%	0.12%
Archaea	0	0	0	0	0	0	0	0	0	0	0.00004%	0
Puccinales	25.61%	0.01%	38.19%	0.01%	41.56%	0.01%	41.46%	0.02%	41.49%	0.01%	43.00%	0.01%
Viridiplantae	52.00%	95.96%	36.61%	95.75%	30.70%	95.72%	30.38%	96.09%	28.56%	95.33%	13.13%	95.22%
Metazoa	0.18%	0.04%	0.46%	0.06%	0.49%	0.07%	0.78%	0.09%	0.29	0.03%	0.32%	0.04%
No-TaxID^*b*^	0.66%	0.46%	0.17%	0.17%	0.27%	0.90%	0.29%	0.03%	0.28%	1.07%	1.93%	0.76%
Unannotated^*c*^	17.47%	2.74%	21.27%	2.89%	23.68%	2.47%	21.88%	2.93%	23.69%	2.44%	18.54%	2.72%

^*a*^ Fungal sequences, excluding those classified under the order Pucciniales.

^*b*^ Sequences with taxonomic IDs not found in the NCBI NR database.

^*c*^ Sequences without significant matches in the NR database.

The composition of bacterial, fungal, and viral transcriptomes was significantly influenced by leaf condition (healthy vs. diseased), infection stage, and their interaction (Fig. 1c; Fig. S1c through e; Table 1; Table S3). Leaf condition was the dominant factor shaping transcriptome composition across all groups. To better visualize these compositional differences, we employed principal coordinate analysis (PCoA) based on Bray-Curtis distance (Fig. 1c; Fig. S1c). The results revealed clear clustering patterns of microbial transcriptomes associated with leaf condition and disease stage. For fungal and viral transcriptomes, the first principal coordinate (PCoA 1) accounted for 46% and 48% of the total variance, respectively, and clearly separated healthy and diseased samples. In contrast, bacterial communities showed their primary separation along PCoA2, which still explained a substantial 33% of the variance. Further analysis showed that bacterial transcriptomes in diseased leaves exhibited the highest Bray−Curtis dissimilarity during the early stage of infection (Stage 1). In contrast, viral communities displayed peak dissimilarity during the final stage of disease progression (Fig. S1d).

### Overview of the composition and differential expression of phyllosphere transcriptomes

**Fig 2:**
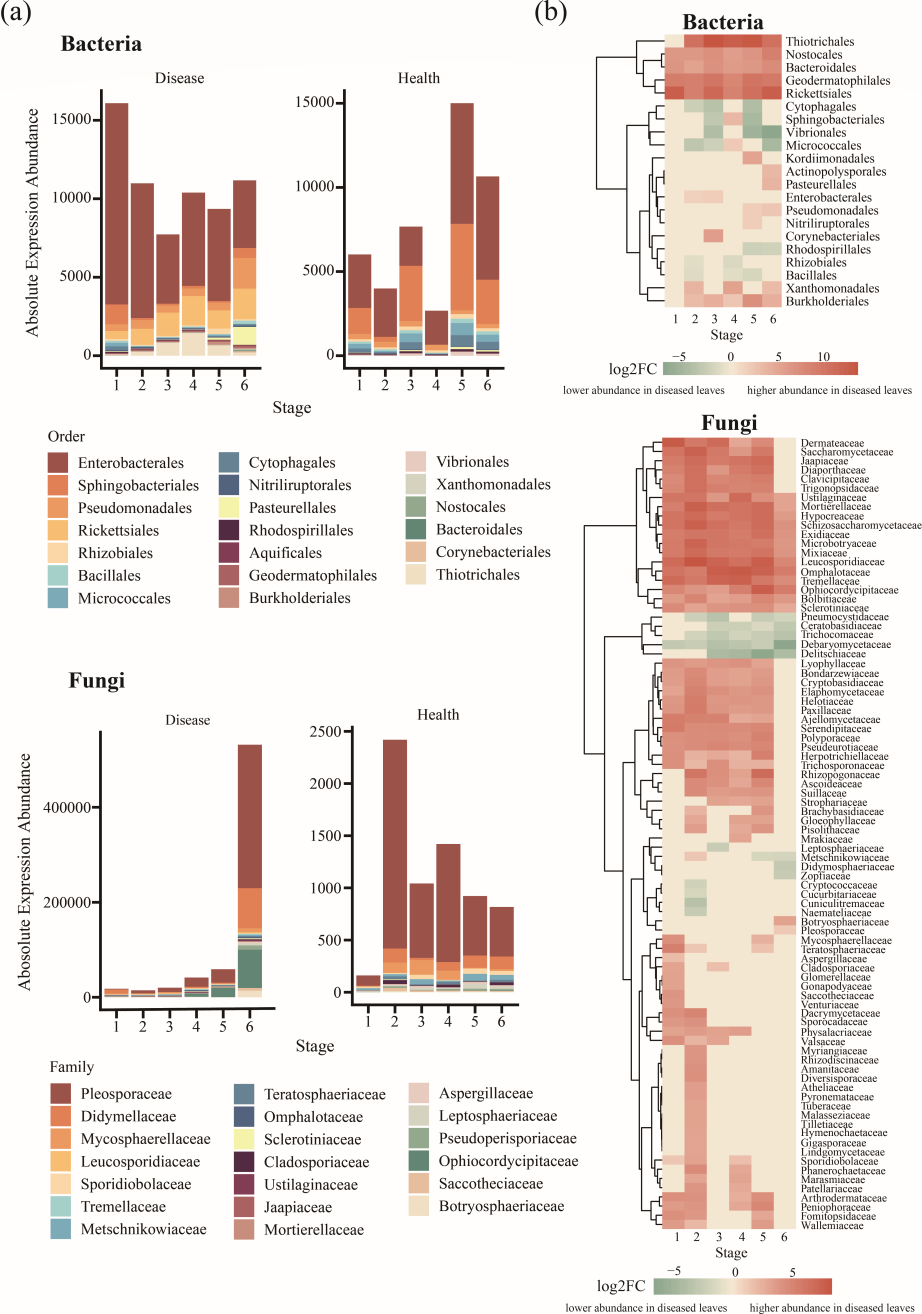
Overview of the composition and differential expression of phyllosphere transcriptomes in crabapple leaves. (**a**) Taxonomic compositions of the bacterial transcriptome at the order level and the fungal transcriptome at the family level, highlighting the top 20 most abundant taxa in both healthy and diseased leaves across different stages of infection. (**b**) Significantly differentially expressed transcripts between healthy and diseased leaves at each stage. Differential expression analysis was performed using a generalized linear model (GLM), and transcripts with *P* < 0.05 (FDR corrected) were considered significant.

We characterized the composition and differential taxonomic profiles of phyllosphere transcriptomes in response to *G. yamadae* infection (Fig. 2; Table 2). The infection substantially reshaped the transcriptomic landscape across bacteria, fungi, viruses, and archaea. In healthy leaves, bacteria dominated the phyllosphere microbiome, consistently exhibiting higher relative expression abundance than the other groups (Table 2). Except at the first stage and the sixth stage, the relative expression abundance of microbial groups generally followed the order: bacteria > fungi > viruses > archaea. Conversely, fungal transcript abundance progressively increased in diseased leaves, peaking at 22.19% in the final stage. Concurrently, the relative expression abundance of bacterial and viral transcriptomes decreased, although bacterial transcripts remained more abundant than viral ones. Archaea were detected only at race levels (0.00004%) during the final diseased stage.

Taxonomical classification of the bacterial transcriptomes at the order level and fungal transcriptomes at the family level revealed notable shifts in microbial composition based on transcriptomic profiles, highlighting the top 20 taxa transcripts by absolute expression abundance (Fig. 2a). In diseased leaves, the dominant bacterial orders included Enterobacterales, Rickettsiales, Pseudomonadales, Thiotrichales, and Sphingobacteriales. In contrast, the most abundant bacterial orders were observed for Enterobacterales, Sphingobacteriales, Micrococcales, Cytophagales, and Pseudomonadales. Several bacterial orders, including Thiotrichales, Nostocales, Bacteroidales, Geodermatophilales, Rickettsiales, Xanthomonadales, and Burkholderiales, showed significant upregulation (*P* < 0.05) in diseased samples compared with healthy leaves (Fig. 2b; Fig. S2). Conversely, Cytophagales, Vibrionales, Rhodospirillales, Rhizobiales, and Bacillales were significantly downregulated in diseased samples (*P* < 0.05).

Overall, the dominant fungal taxa in healthy leaves exhibited lower transcript abundance than their bacterial counterparts (Fig. 2a). Moreover, fungal transcripts showed greater variation in response to pathogen disturbance, with a more pronounced increasing trend over time. Notably, Pleosporaceae, Ophiocordycipitaceae, and Didymellaceae exhibited a sharp increase at the final diseased stage. In addition, Dermateaceae was notably upregulated in diseased leaves and clustered together in the hierarchical analysis (Fig. 2b). Unlike bacterial communities, which included both up- and down-regulated taxa, fungal transcripts generally showed increased expression during rust infection, with relatively fewer taxa downregulated. Interestingly, certain bacterial taxa exhibited stage-specific differential expression patterns—for instance, Sphingobacteriales and Micrococcales were significantly upregulated at the third diseased stage but downregulated during the early and late stages. No similar temporal trends were observed among fungal taxa.

### Changes in phyllosphere gene expression and functional profiles during *G. yamadae* infection

**Fig 3:**
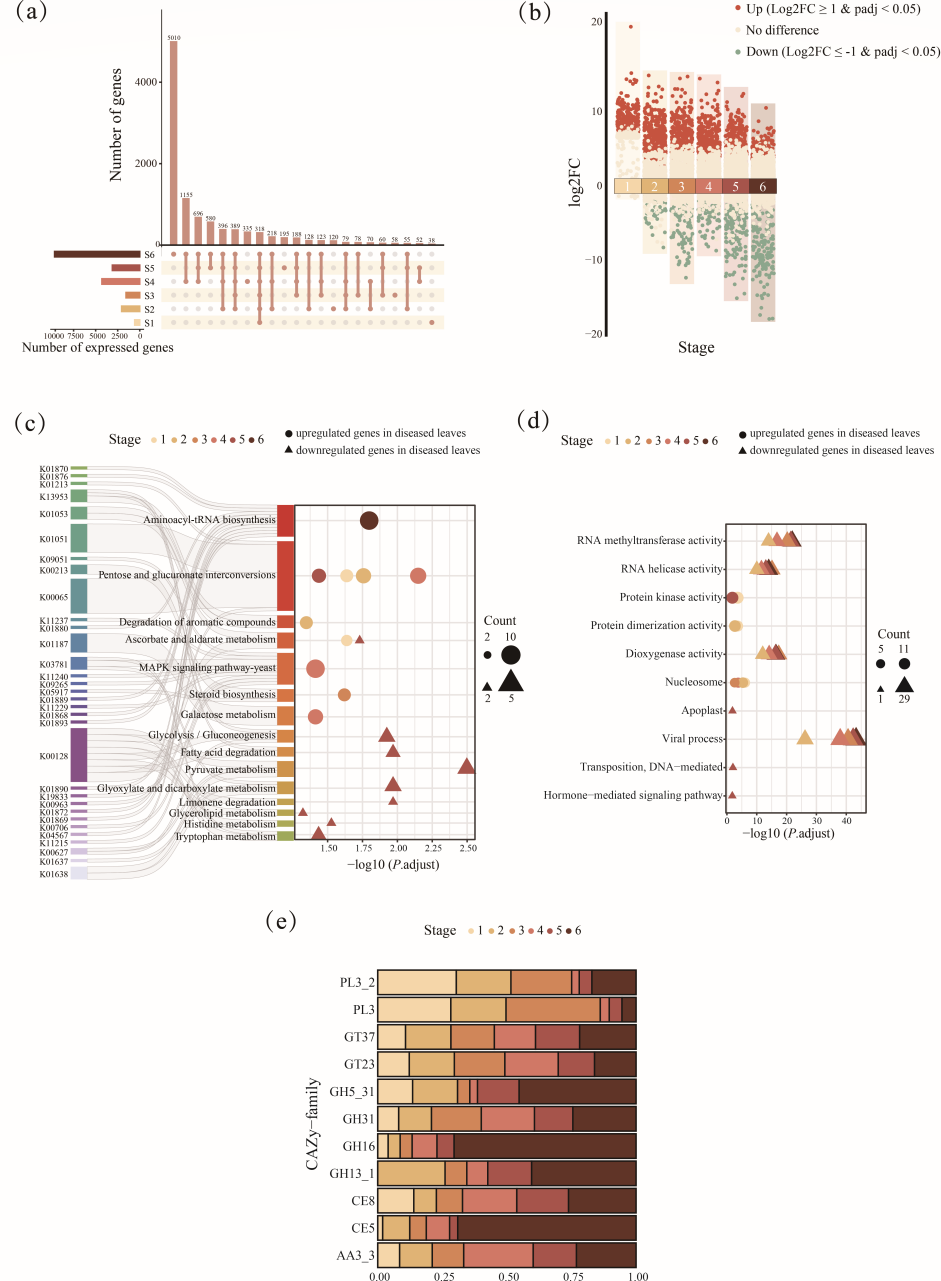
Expression profiling and functional annotation of phyllosphere transcripts in crabapple leaves during *Gymnosporangium yamadae* infection. (**a**) UpSet plot showing the overlap and uniqueness of expressed genes across six disease stages. The top 22 intersecting gene sets are displayed. (**b**) Volcano plots of differentially expressed genes (DEGs) between diseased and healthy leaves at each stage of infection. Red dots indicate upregulated genes in diseased samples (log_2_fold change ≥ 1, adjusted *P*-value < 0.05), green dots indicate downregulated genes (log_2_fold change ≤ −1, adjusted *P*-value < 0.05), and the yellow dots denote genes with no significant difference. (**c, d**) The KEGG pathway (**c**) and GO term (**d**) enrichment analyses of DEGs using the hypergeometric test. Significance is shown as log₂-transformed *P*-values (FDR-corrected). Symbol sizes represent the number of enriched genes; triangles indicate downregulation, and circles indicate upregulation. (**e**) Relative expression abundance of significantly differentially expressed carbohydrate-active enzymes (CAZy) families across different rust disease stages.

To determine the gene expression dynamics across *G. yamadae* infection, we quantified and visualized the number of expressed unigenes assigned to bacteria, fungi, viruses, and archaea (Fig. 3a). Both the total number of expressed genes and the number of stage-specific genes, defined as genes uniquely expressed at a particular stage, were lowest at the initial stage and peaked at the final stage of infection. To identify differentially expressed genes (DEGs), pairwise comparisons were performed between transcriptomes from diseased and healthy crabapple leaves at each stage. Transcripts with significant differential expression (log_2_fold-change ≥ 1 or ≤ −1, adjusted *P* value < 0.05) were categorized as DEGs (Fig. 3b; Table 3; Table S4). As rust disease progressed, gene expression increased markedly, with upregulated genes consistently outnumbering downregulated ones at each stage of infection.

**TABLE 3:** The results of differential gene expression analysis in crabapple phyllosphere at each stage

Stage	Upregulated^*a*^	Downregulated^*b*^	Total
1	194	0	194
2	339	40	379
3	298	78	376
4	382	67	449
5	226	129	355
6	306	168	474

^*a*^ Genes that were significantly upregulated in diseased leaves (log_2_fold change ≥ 1, adjusted *P*-value < 0.05) compared with healthy leaves were classified as upregulated genes.

^*b*^ Genes that were significantly downregulated in diseased leaves (log_2_fold change ≤ −1, adjusted *P*-value < 0.05) compared with healthy leaves were classified as downregulated genes.

To investigate how rust infection and disease progression influence the functional attributes and activities of the crabapple phyllosphere microbiota, we functionally annotated the targeted transcripts (Fig. 3c through e; Fig. S3 and S4). Of the target unigenes identified, 12,360 (46.3%), 14,055 (52.7%), and 776 (2.9%) were assigned to the Kyoto Encyclopedia of Genes and Genomes (KEGG), Gene Ontology (GO), and Carbohydrate-Active enZYmes (CAZy) databases, respectively (Tables S5 to S7). After manually filtering out annotations unrelated to phyllosphere microorganisms (e.g., those associated with humans or animals), we performed KEGG and GO enrichment analysis on DEGs at each stage to identify significantly enriched pathways (Fig. 3c and e). Overall, microbial transcripts in diseased leaves were predominantly enriched in pathways related to primary metabolism. Trend analysis of KEGG functional categories indicated a general increase in transcript abundance across most pathways during disease progression (Fig. S3). Although global metabolism-related expression patterns in diseased leaves showed limited temporal differentiation, certain specific pathways exhibited distinct stage-dependent enrichment. For example, the pentose and glucuronate interconversions pathway was significantly enriched across multiple stages (Fig. 3c). Conversely, transcripts associated with RNA methyltransferase activity, RNA helicase activity, dioxygenase activity, and viral process were significantly downregulated at all disease stages compared with healthy leaves (Fig. 3d). Specifically, at the onset of the disease, certain transcripts related to carbohydrate metabolism were notably enriched, such as those involved in ascorbate and aldarate metabolism category (Fig. 3c). Simultaneously, pathways involved in the degradation of aromatic compounds were active during the second stage of infection. Transcripts associated with cellular components affecting gene expression and regulation, such as nucleosome, as well as those related to protein kinase and dimerization activity, were significantly upregulated in the early stages of rust disease (Fig. 3d). As lesions expanded, pathways including steroid biosynthesis, galactose metabolism, and MAPK signaling were significantly enriched, alongside sustained activity of nucleosome (Fig. 3c and d). Interestingly, a few categories showed significant upregulation in the late stages of rust disease, such as the aminoacyl-tRNA biosynthesis pathway related to translation, and genes associated with protein kinase activity also exhibited significant enrichment in the latest stage of the disease progression. In addition to the pathways and molecular functions mentioned above, which were mostly downregulated across all diseased stages, certain functions were significantly downregulated only in the late stages of rust disease, including carbohydrate metabolism, lipid metabolism, amino acid metabolism, and apoplastic activity (Fig. 3c). Notably, some pathways exhibited temporal patterns, with the ascorbate and aldarate metabolism pathway enriched in the first stage of *G. yamadae* infection but suppressed in the fifth stage.

**Fig 4:**
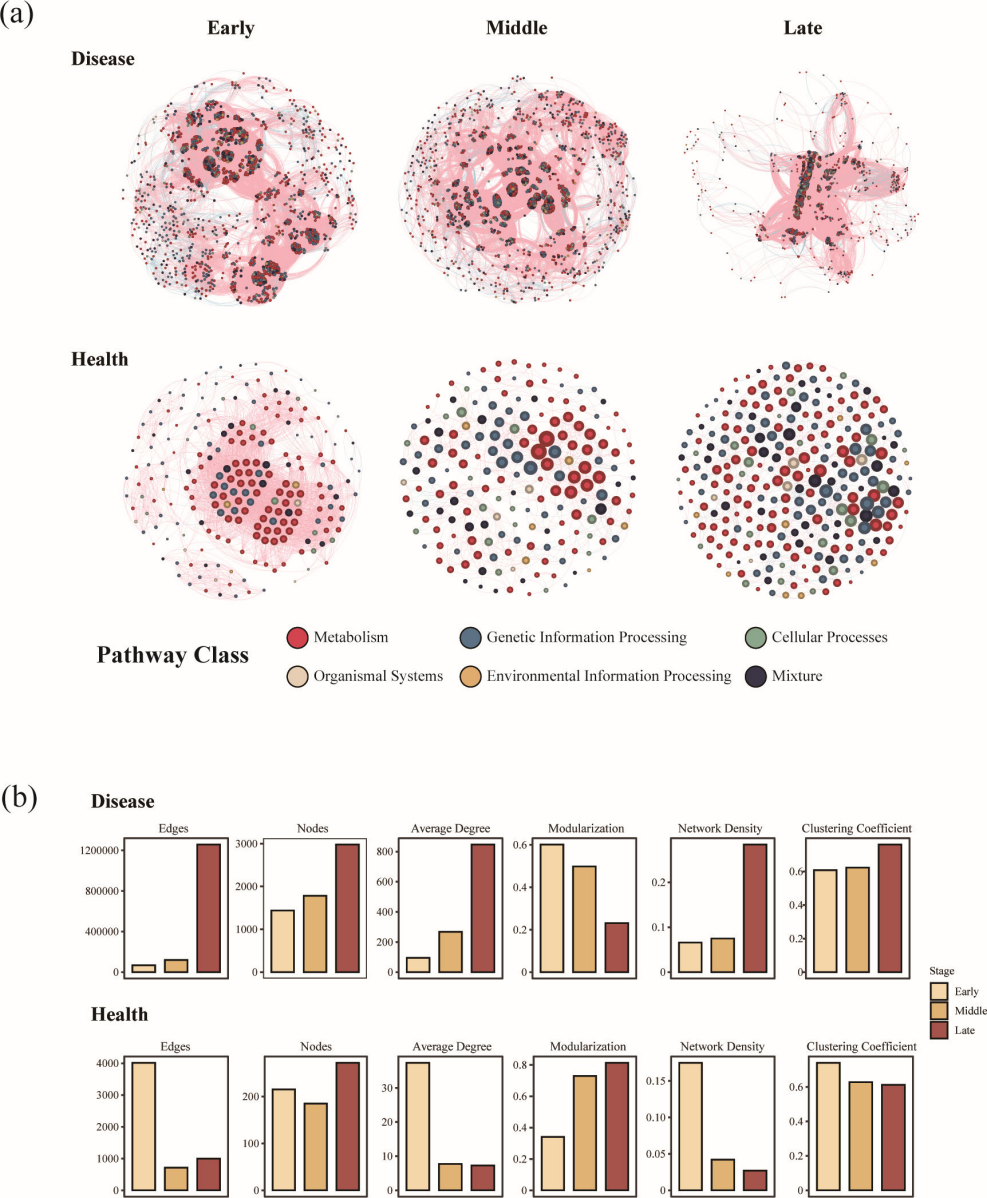
The co-occurrence networks of phyllosphere microbiome functional genes in diseased and healthy samples at different stages of rust disease. (**a**) Co-occurrence networks of functional genes in diseased and healthy samples at early, middle, and late stages of infection. The nodes in the networks are colored according to functional annotations derived from the KEGG database. Positive correlations between genes are represented by red edges, whereas negative correlations are indicated by blue edges. (**b**) Topological properties of the functional gene co-occurrence networks for both diseased and healthy samples across different stages of disease progression.

To gain a more detailed resolution of specific functions associated with metabolism-related pathways, we annotated transcripts using the CAZy database (Fig. 3e). Similar to the KEGG analysis results, the diversity of KEGG orthology (KO) and CAZy families showed similar temporal patterns, and *G. yamadae* infection also led to an increased expression of all CAZy families (Fig. S4a and b). After identifying differentially expressed CAZymes across various disease stages, we focused on 11 CAZy families actively involved in regulation (Fig. 3e; Fig. S4c). Remarkably, certain glycoside hydrolases, potentially related to fungal cell wall component degradation, exhibited increased activity in diseased leaves. These include enzymes involved in the degradation of chitin (GH5), glucans (GH5, GH16, and GH13), and mannans (GH5 and GH31). The relative expression abundance of these glycoside hydrolases (GH5, GH16, and GH13) increased as the lesions expanded and became more enriched in the late stages of rust disease (Fig. 4c). Additionally, CAZy families that may interact with the host plant were also enriched in the infected leaves at different stages, including enzymes that participated in the degradation of plant cell wall components such as pectin (PL3 and CE8), cellulose (AA3), lignin (AA3), and xylan (CE5) (Fig. 4c; Fig. S4c). Among these, the AA3 and CE5 families were upregulated in early and middle stages of rust disease, similar to the patterns of glycoside hydrolases. In contrast, PL3 and CE8 families were upregulated in the late stages (Fig. S4c).

### Dynamic changes in the complexity of phyllosphere microbiome functional co-occurrence networks as lesions expanded

To assess how lesion expansion impacts interactions among functional genes in the phyllosphere, we conducted co-occurrence network analysis and calculated topological properties for both healthy and diseased leaves at various stages (Fig. 4). In all groups, functional genes related to microbial metabolism were the most predominant category (Fig. 4a). The infection of *G. yamadae* altered the complexity of the phyllosphere co-occurrence networks, with network complexity indices showing opposite trends between healthy and diseased samples. Specifically, diseased leaves exhibited higher values in the number of nodes, number of edges, and average degree indices compared with healthy leaves, with these indices gradually increasing as lesions expanded (Fig. 4b). The modularization index, initially higher in diseased during the early stages of infection than in healthy samples, decreased progressively throughout the rust disease progression, eventually falling below the levels seen in healthy leaves by the middle and late stages of infection. In contrast, network density and clustering coefficient indices were higher in healthy leaves at early stages but elevated in diseased samples as the infection advanced to the middle and late stages.

### Contribution of phyllosphere functional genes to the pathogenesis of crabapple rust disease

**Fig 5:**
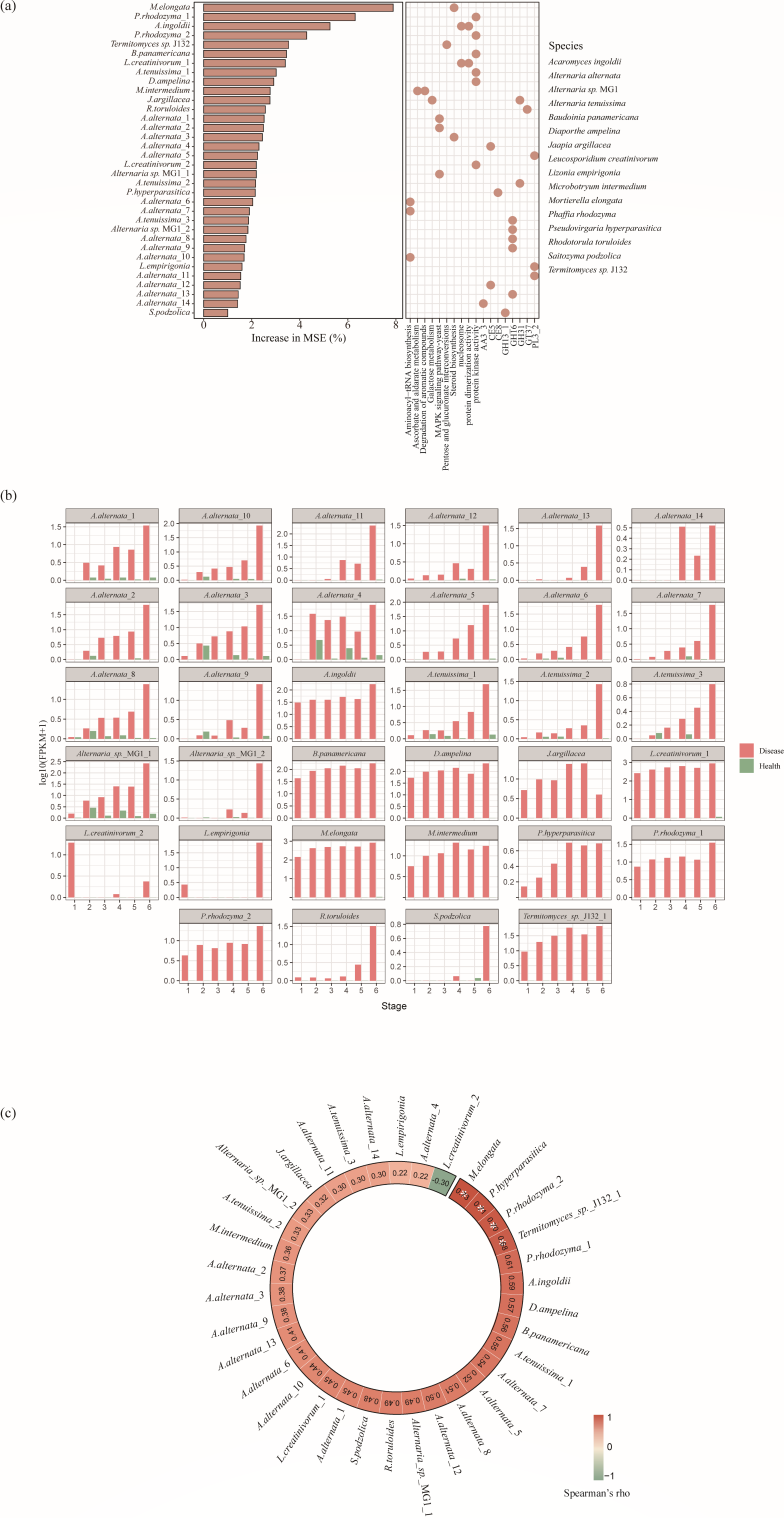
Key phyllosphere microbial functional genes associated with the severity of crabapple rust disease and their correlation with host metabolites. (**a**) Importance ranking of phyllosphere microbial functional genes associated with disease severity, identified using random forest modeling. The bar plot (left) shows the mean percentage increase of mean square error (MSE) when each gene is permuted, with higher values indicating greater importance in predicting Pucciniales (pathogen) abundance. The dot plot (right) illustrates the associated functional categories of these genes; the *x*-axis represents the functional annotations (e.g., pathways or enzyme types), and the *y*-axis lists gene identifiers. All functional genes shown were statistically significant in the model (*P* < 0.05). (**b**) Expression profiles for key functional genes across different disease stages and conditions. (**c**) Spearman’s correlation between the expression of selected functional genes and the abundance of host-derived flavonoid glycosides within diseased leaf samples. Asterisks indicate the statistical significance: *P* < 0.05, FDR corrected.

To identify transcripts actively involved in the pathogenesis of crabapple rust disease, we conducted a random forest analysis focusing on transcripts related to *G. yamadae* (order Pucciniales) and their contribution to disease progression (Fig. 5a and b; Table S8). In addition, we examined the correlations between these transcripts and flavonoid glycosides, which have been identified as host-derived metabolites previously implicated in lesion expansion (8) (Fig. 5c).

Among the 34 transcripts significantly predictive of Pucciniales abundance (*P* < 0.05), *Alternaria alternata* emerged as the most predominant species, with all associated transcripts showing higher expression levels in diseased samples compared with healthy ones (Fig. 5a and b). These key transcripts were primarily involved in several critical biological processes and pathways: the MAPK signaling pathway, aminoacyl-tRNA and steroid biosynthesis, as well as the production of CAZy families that target plant cell wall components. Specifically, enzymes that degrade pectin (PL3), xylan (CE5), and cellulose (AA3) were linked to this functional activity. Additionally, *Pseudovirgaria hyperparasitica* contributed significantly to the production of pectin-degrading enzymes (CE8), which facilitate the plant cell wall breakdown (Fig. 5a). This transcript was exclusively expressed in diseased samples and showed a strong positive correlation with flavonoid glycosides abundance in the leaves (Fig. 5b and c). Transcripts involved in the steroid biosynthesis process were primarily attributed to *Mortierella elongata*. These transcripts not only ranked as the most important contributor to changes in Pucciniales abundance but also correlated positively with flavonoid glycoside levels (Fig. 5a through c). Additional functional roles were assigned to the following other species: *Microbotryum intermedium* in aromatic compounds degradation and ascorbate/aldarate metabolism; *Jaapia argillacea* in galactose metabolism; and *Termitomyces* sp. J132, which was significantly correlated with flavonoid abundance, was involved in pentose and glucuronate interconversions. Interestingly, several transcripts associated with fungal cell wall degradation also showed significant importance in the model, including genes involved in the degradation of glucans (e.g., from *Saitozyma podzolica* and *Alternaria tenuissima*) and mannans (e.g., from *Alternaria tenuissima* and *Jaapia argillacea*). These transcripts were generally more highly expressed in diseased samples than in healthy ones, underscoring the critical role of microbial interactions and enzymatic activities in the pathogenesis and progression of crabapple rust disease.

## DISCUSSION

### Rust disease shapes phyllosphere microbiome assembly in crabapple

The assembly and composition of the plant microbiome are intricately linked to plant health. Pathogen infections frequently drive substantial changes in microbial community structure and function, which, in turn, can modulate host resistance or susceptibility across diverse plant-pathogen systems (3). In this study, the observed overall increase in microbial richness in diseased crabapple leaves compared to healthy ones suggests that a broad range of microbial taxa actively respond to *G. yamadae* infection (8).

An intriguing observation in our study was the negative correlation between bacterial and fungal transcriptome diversity in response to pathogen infection (Fig. 1a and b), suggesting a functional complementarity or inhibitory relationship in their transcriptional patterns, potentially driven by pathogen-induced shifts in the phyllosphere microbiome (40). As *G. yamadae* colonizes the leaf surface and expands lesions, selective pressures may promote the proliferation and enhanced gene expression of certain fungal species, potentially suppressing the transcriptional activity of bacterial communities (41). Further support for this competitive dynamic is provided by changes in community evenness: as lesions expanded, bacterial species displayed more uniform expression abundance (increased evenness), whereas the fungal species showed greater divergence in expression abundance (decreased evenness) (Fig. S1b). We postulate that this divergence in fungal community evenness may be induced by pathogen invasion, which perturbs the ecological balance of the phyllosphere microbiome. This allows certain epiphytic fungi, particularly those associated with the pathogen, to rapidly colonize and infect the compromised host tissues, thereby putting bacterial communities at a competitive disadvantage, especially in terms of nutrient acquisition (8). This hypothesis is reinforced by the observed patterns in the relative expression abundance across the microbial groups in the phyllosphere: bacteria consistently dominated across all stages in healthy leaves, whereas fungi progressively emerged as the predominant microbial group in diseased leaves, with their relative abundance peaking as the lesions expanded (Fig. 2a). These findings are not entirely consistent with previous studies of phyllosphere microbiome under *G. yamadae* infection based on amplicon sequencing, which reported a bacteria-dominated response to the disease (8, 36). However, such discrepancies may reflect methodological and biological differences between approaches. Although amplicon sequencing captures changes in microbial composition and relative abundance, metatranscriptomics reveals functionally active microbial populations (6). Our results indicate that fungal taxa exhibit more pronounced transcriptional responses during disease progression, likely reflecting their expanded functional roles during *G. yamadae* infection. These findings underscore the increasing importance of fungi—both opportunistic pathogens and symbiotic microbes—in the pathogenesis process (42).

### Shifts in microbial community composition and functional groups

PCoA results revealed that microbial community composition in the phyllosphere was strongly influenced by leaf condition, with distinct microbial signatures found in diseased leaves versus healthy leaves (Fig. 1c; Fig. S1c and e). Interestingly, during the early and middle stages of infection, bacterial diversity was lower than in healthy leaves at corresponding stages; however, in the late stages of infection, bacterial diversity in the diseased leaves surpassed that in healthy leaves (Fig. 1a). This suggests an adaptive restructuring of the microbial community over the course of the infection, where specific taxa may have been displaced or outcompeted initially, but later stages of infection provide new ecological niches (43). The infection of *G. yamadae* significantly altered the composition of the phyllosphere microbial community, and the profiles of expression abundances revealed notable shifts (Fig. 2; Table 2). Although the dominant bacterial taxa at the order level were relatively similar between healthy and diseased leaf samples, certain members were notably upregulated or downregulated in response to pathogen infection (Fig. 2; Fig. S2). For example, the orders Nostocales and Rickettsiales were significantly upregulated, whereas Bacillales was significantly downregulated in diseased leaves. This suggests that pathogen infection triggers a distinct microbial response in terms of gene expression, likely reflecting shifts in microbial activity associated with disease progression. In the fungal community, the family *Pleosporaceae* was the dominant group in both diseased and healthy leaves. In addition, the expression abundance of fungal transcripts gradually increased under pathogen infection and peaked at the final stage (Table 2), with most fungal transcripts generally upregulated in response to infection (Fig. 2)—a trend more pronounced than that observed in bacterial communities. This suggests a greater involvement of fungi in the host response to pathogen infection, possibly through directly or indirectly exacerbating or modulating the development of the pathogen (44). However, the precise roles of these microorganisms in the rust disease system require further validation through isolation, cultivation, and functional assays.

### Functional attributes and microbial network complexity

Functional analysis revealed that *G. yamadae* infection significantly altered both functional attributes and co-occurrence patterns within the phyllosphere microbiome. Notably, the functional co-occurrence networks in infected leaves exhibited markedly greater complexity compared with those in healthy counterparts (Fig. 4), aligning with prior taxonomic observations of more intricate microbial community structures under rust infection (8). This parallel restructuring at taxonomic and functional levels indicates that the microbiome’s adaptive response to pathogen invasion involves not only compositional alterations but also profound reorganization of microbial interactions and functional connectivity (45) while echoing findings from other plant-pathogen systems where pathogen pressure elevates microbial network complexity (46, 47). For example, citrus plants infected with *Diaporthe citri* and chili peppers suffering from *Fusarium* wilt displayed more intricate co-occurrence networks in their phyllosphere and rhizosphere microbiomes, respectively, compared with uninfected controls (16, 26). These more complex networks have been proposed to arise from stress-induced restructuring of microbial associations, which may reflect ecological processes that promote greater community stability and functional redundancy (46, 47). In our study, the increased complexity of functional co-occurrence networks in *G. yamadae*-infected samples likely indicates enhanced interdependence among microbial functions under pathogen-induced perturbation. This restructuring may act as a buffering mechanism, where microbial communities reorganize to either mitigate the pathogenic impact or sustain critical functions necessary for host resilience (48). We hypothesize that this complexity reflects enhanced interactions that present protective or antagonistic responses against the pathogen. Further support for this interpretation is provided by changes in key topological metrics, particularly modularity, which reflects the degree of compartmentalization or specialization within networks (49). During the early stages of *G. yamadae* infection, infected leaves exhibited higher modularity than healthy ones (Fig. 4b), suggesting more structured and potentially functionally cohesive microbial subgroups. This compartmentalization may reflect microbial consortia co-regulating key defense-related functions, potentially enhancing the plant’s ability to respond to the pathogen (16, 49–51). However, as the disease progressed, this stable co-regulation is disrupted, becoming less pronounced compared with healthy leaves, which evolved more stable functional networks over time (47). Despite these insights, it is important to interpret network-based inferences with caution. Co-occurrence networks—although useful for detecting correlations—do not establish causality or confirm direct ecological interactions (52). Therefore, although our findings suggest that *G. yamadae* infection induces substantial restructuring of microbial functional interactions, further experimental validation (e.g., synthetic community experiments or perturbation assays) is required to determine the ecological and functional significance of these observed network dynamics (46, 52, 53).

### Opportunistic microbe-mediated metabolic adaptation and enzyme regulation

Functional analysis provided crucial insights into the mechanisms underlying the dynamic reorganization of the phyllosphere microbial community during *G. yamadae* infection. A key observation was the significant upregulation of energy metabolism pathways in infected samples, relative to healthy controls (Fig. S3). Specifically, DEGs were enriched in carbohydrate metabolism, with sustained activation of the pentose and glucuronate interconversion pathway across disease stages (Fig. 3c). These pathways support the decomposition of complex plant-derived polysaccharides (e.g., pectin), potentially enabling microbes access to carbon in the nutrient-scarce phyllosphere (54, 55). This metabolic adaptation is further supported by the upregulation of plant cell wall-degrading enzymes (e.g., pectin lyase [PL3], carbohydrate esterase [CE8], and acetylxylan esterase [CE5]) within diseased samples, as evidenced by CAZy family analysis (Fig. 4c). These enzymes likely enable phyllosphere microbes to exploit exogenous carbon compounds released from host tissue damage during lesion formation (24). Notably, *Termitomyces* sp. J132, a microbe exclusively detected in diseased samples, expressed transcripts involved in the pentose and glucuronate interconversions pathway and was identified as a strong predictor of Pucciniales abundance in the random forest model (Fig. 5a and b). Its transcriptional activity was positively correlated with the accumulation of flavonoid glycoside—host-derived metabolites associated with lesion expansion (8, 37–39). Similarly, other taxa, including *Alternaria alternata* and *Pseudovirgaria hyperparasitica,* also expressed plant cell-wall degrading enzymes and correlated strongly with flavonoid levels, suggesting opportunistic colonization of compromised host tissues (56, 57). These microbes likely benefit from the metabolite-rich lesion microenvironment, contributing indirectly to disease progression by facilitating further tissue breakdown (58). Collectively, they may constitute a “pathobiome” consortium that co-opts host-derived resources and exacerbates disease symptom severity (6, 20–22).

In addition to metabolic adaptation, the phyllosphere microbiota may also contribute to detoxifying host defense compounds. Previous untargeted metabolomic analysis of the host leaves under identical experimental conditions revealed elevated levels of aromatic compounds, which are typically involved in plant defense responses (8). Correspondingly, early-stage disease samples showed microbial enrichment in pathways related to aromatic compound degradation functions, indicating an active microbial role in detoxifying or repurposing these inhibitory compounds (59). This may reduce the phytotoxic impact of these metabolites, creating a more permissive environment for both the pathogen and coexisting microbes. Simultaneously, the ascorbate and aldarate metabolism pathways were significantly enriched during the early infection stages (Fig. 3c). Ascorbate plays a central role in redox homeostasis and stress mitigation in plants; therefore, microbial engagement in its metabolism may reflect a coordinated microbial response to oxidative stress within the pathogen-altered phyllosphere environment (60, 61). These adaptations reflect microbial resilience and a capacity for functional reprogramming in response to a rapidly changing habitat (16, 62, 63).

The phyllosphere microbiome may also engage in multifaceted roles that influence plant health, including contributions to plant defense mechanisms (3, 27). Plants often recruit beneficial microorganisms with antagonistic capabilities against pathogens, incorporating them into defense responses (34, 64, 65). For example, sugar beet roots infected with *Rhizoctonia solani* enrich for *Chitinophagaceae* and *Flavobacteriaceae*, which secrete fungal cell wall-degrading enzymes that suppress pathogen infection (17). Similarly, our study identified active secretion of fungal cell wall-degrading enzymes (GH5, GH16, GH13, and GH31), targeting chitin, glucan, and mannan during early to middle stages of rust infection (Fig. 3e; Fig. S14c) (66). This supports the notion that members of the phyllosphere microbiome are mobilized to counteract the pathogen through enzymatic degradation of its structural components (17, 26, 67). Random forest modeling identified key taxa associated with these antifungal functions, including *Jaapia argillacea* and *Saitozyma podzolica* (Fig. 5a). *S. podzolica*, a known plant growth-promoting (PGP) yeast, has been previously reported to inhibit pathogens like *Fusarium oxysporum* through secretion of fungal cell wall-degrading enzymes and antifungal metabolites (68). In our study, transcripts from *S. podzolica* exhibited high transcriptional activity during late infection stages (Fig. 5b), suggesting a delayed yet pronounced defensive activation. This temporal pattern may indicate that the antagonistic activity of beneficial microbes is tuned to host stress intensity or disease phase, illustrating adaptive microbial behavior in response to dynamic plant conditions (69–71).

Notably, the late activation of putative protective functions in *S. podzolica* contrasts with the early opportunism of saprophytic taxa that exploit nutrient leakage from damaged tissues. This temporal niche differentiation highlights the functional diversity and ecological complexity of the phyllosphere microbiome during disease (8, 28, 36). Although *S. podzolica* may contribute to pathogen suppression or tissue recovery in late infection, its mechanistic role and ecological impact within the community require experimental validation through targeted functional assays, synthetic community inoculations, and microbe-host interaction studies (19, 24, 27, 72).

### Conclusion

Overall, our findings reveal a complex and dynamically reprogrammed functional landscape in the phyllosphere microbiome throughout the progression of carbapple rust disease, shaped substantially by opportunistic microbes (3, 16, 62, 63). Key microbial activities included the breakdown of pectin from damaged host tissues and the potential detoxification of plant defense compounds through aromatic metabolite degradation (24, 59). In the later stages of disease, we also observed increased microbial potential for fungal cell wall decomposition. These results underscore the multifaceted and interconnected functional roles played by the phyllosphere microbiome during plant-pathogen interaction networks (24). The observed microbial adaptability—ranging from nutrient acquisition and detoxification to antagonistic activity—highlights a collective metabolic resilience under stress-induced conditions. Beyond elucidating microbial dynamics in the rust-crabapple pathosystem, this study provides a functional framework for future targeted investigations into microbial contributions to plant health and disease outcomes.

## MATERIALS AND METHODS

### Sample collection

Crabapple (*Malus* ‘Kelsey’) leaves were collected from trees located at the south gate of Olympic Park, Beijing (40 °N, 116.38 °E) between June and September 2021. This sampling period corresponds to key developmental stages of *G. yamadae* in the aecial host, encompassing the formation and maturation of both spermogonia and aecia. For each stage, healthy and diseased leaves were collected in parallel from the same individual trees to control for host genetic variability. Three biological replicates were sampled per condition and disease stage. Healthy leaves were defined as those without any visible symptoms of disease, pest infestation, or physical damage. Diseased leaves were selected based on the presence of well-developed rust lesions, with no signs of co-infection or abiotic stressors. Diseased stages were categorized according to the macroscopic characteristics of the rust lesions. The proportion of lesion coverage at each stage is described in detail in our previous study (8). The leaf samples were immediately transported to the laboratory on dry ice and stored at −80°C until RNA extraction.

### RNA extraction and metatranscriptomic sequencing

Leaf tissues were ground into a fine powder using RNase-free mortars and pestles with liquid nitrogen. For diseased samples, grounding was performed at the lesion–healthy interface, ensuring that lesions covered approximately half of the selected area. Total RNA was extracted from the phyllosphere using the Fecal RNA Extraction Kit (Majorbio, Shanghai, China), following the manufacturer’s procedure. The integrity and concentration of the extracted RNA were measured with a NanoDrop 2000 spectrophotometer (Thermo Scientific, MA, USA) and an Agilent 5300 Bioanalyzer (Agilent Technologies, Palo Alto, CA, USA). For sequencing, 200 ng of each sample’s RNA was used for library preparation with the Illumina Stranded mRNA Prep, Ligation (Illumina, San Diego, CA, USA). Paired-end sequencing was carried out on the Illumina Novaseq 6000 at Majorbio Bio-Pharm Technology Co., Ltd. (Shanghai, China).

### Bioinformatics and statistical analysis

#### Data preprocessing and quality control

Raw sequencing data were processed to ensure high quality and minimize host and non-target contamination (Table S9). Quality filtering was performed using fastp v0.19.6 (73), which included adapter trimming at both 3′ and 5′ ends of reads. Reads were discarded if they were shorter than 50 bp, had an average Phred quality score below 20, or contained ambiguous bases (N). To eliminate host-derived sequences, reads were mapped to four Malus species genomes*—Malus domestica* (GCF_002114115.1), *Malus baccata* (GCA_006547085.1), *Malus sylvestris* (GCF_916048215.2), and *Malus sieversii* (GCA_020795835.1) using BWA (74), and any matching reads were removed. To further eliminate non-target sequences, ribosomal RNA sequences were further filtered out using SortMeRNA (75) to exclude potential rRNA contamination. Cleaned reads were assembled *de novo* using Trinity v2.2.0 (76), with transcripts with lengths ≥ 300 bp retained for downstream analysis (77, 78). Open reading frames (ORFs) of all assembled transcripts were predicted using TransGeneScan software to obtain amino acid sequences for subsequent functional annotation and downstream analyses (79). Redundant transcripts were clustered using CD-HIT v4.6.1 (80), applying a sequence identity threshold of 95% and a minimum coverage of 90%. The longest representative sequence from each cluster was designated as a unigene for downstream annotation and analysis.

#### Taxonomic annotation

The assembled unigenes were translated into amino acid sequences and annotated using DIAMOND v0.8.35 (81) against the NCBI NR (non-redundant) protein database. The top hit for each unigene was selected based on an e-value cutoff of 1e-5 (30, 82, 83). Transcript abundance was estimated using the RSEM v1.3.2 (84), which generated read counts prior to calculating FPKM (fragments per kilobase of transcript per million mapped reads) values for each sample. These values enabled cross-sample comparisons of microbial activity in the phyllosphere microbiome. To focus specifically on non-host and target microbial communities, sequences taxonomically assigned to Viridiplantae (42.9%), Metazoan (0.2%), and Pucciniales (16.5%), along with unassigned sequences (21.3%), were removed prior to downstream microbial community analyses (Table S9).

#### Diversity analysis

Based on transcript expression levels and taxonomic annotations, the phyllosphere microbial community was categorized into bacterial, fungal, viral, and archaeal transcriptomes. Archaea were excluded from subsequent diversity and structural analyses due to their low abundance. Transcript abundance at the species level was calculated by summing the FPKM values of all unigenes annotated to each species (Table S1). Transcriptional alpha diversity indices, including species richness, Shannon diversity, and Pielou’s evenness, and beta diversity using Bray-Curtis dissimilarity were calculated using the vegan package (85) in R from these species-level FPKM values. Principal coordinate analysis (PCoA) was conducted to visualize beta diversity patterns across disease stages and leaf health conditions. Statistical comparisons of alpha diversity indices between diseased and healthy leaves were performed using one-way ANOVA followed by Tukey-HSD *post hoc* test or alternatively Kruskal-Wallis test followed by Wilcoxon’s rank-sum test when data did not meet normality assumptions via the multcomp package (86). The correlation between bacterial and fungal Shannon indices was evaluated using generalized linear models (GLM) with the ggpmisc package (87). Permutational multivariate analysis of variance (PERMANOVA) was applied to test for significant differences in community composition between sample groups using the vegan package (85). Relative transcript expression abundance for microbial groups and taxa was derived from their FPKM values, and significantly differentially expressed taxa were identified based on comparisons of absolute expression levels across treatments.

#### Differential expression and functional analysis

To identify differentially expressed genes (DEGs) and explore functional dynamics, the unigene expression matrix was filtered to retain genes consistently expressed across all three biological replicates per treatment. Genes expressed across different disease stages were visualized using the UpSetR package (88). Differentially expressed genes, transcripts, and carbohydrate-active enzymes (CAZymes) were identified using the DESeq2 package (89) based on read counts, with pairwise comparisons performed between diseased and healthy samples at each stage. Pairwise comparisons of CAZy family expression profiles were made between adjacent sampling stages, applying a generalized linear model (GLM) approach with a significance threshold of *P* < 0.05 after false discovery rate (FDR) correction.

Predicted protein sequences were aligned against the KEGG database using BLASTP v2.2.31 (90), and functional annotation was performed based on the alignment results using KOBAS v2.0 (91). GO annotation of the gene set was conducted using blast2go (92), and hmmscan v3.3.2 was employed to annotate against the CAZy database (93). For all annotations, the e-value cutoff was set to 1e−5, and the best hit for each sequence was selected. The dynamic abundance of KEGG classifications and CAZy families was evaluated using normalized FPKM values, with statistical differences assessed via a one-way ANOVA test. To identify enriched functional categories among DEGs, GO and KEGG enrichment analyses were conducted using the clusterProfiler package (94), employing the hypergeometric test (a statistical method used to assess whether a specific functional category is significantly enriched among DEGs compared with a background gene set) and adjusting for multiple testing via Benjamini-Hochberg FDR correction. The annotated KEGG and GO gene sets served as the background population for enrichment calculations.

Functional co-occurrence network analysis was performed by calculating Spearman correlations of functional genes using the Hmisc package (95). Network topological properties (e.g., modularity, degree) were computed and visualized using Gephi (96) to evaluate functional interaction complexity over disease progression. To identify microbial functions predictive of pathogen dynamics, a random forest model we constructed used the rfPermute package (97). The relative abundance of Pucciniales was set as the response variable, and microbial functional gene expression levels were used as predictors. Host metabolite data (specifically, flavonoid glycoside concentrations) were derived from our previous study (8), and Spearman correlations were calculated between targeted transcript expression and metabolite abundance using the Hmisc package (95).

## Data Availability

All the raw sequencing data from this project are available in the NCBI Sequence Read Archive (SRA) database under BioProject PRJNA1191162. Biosample accessions are SAMN45069823–SAMN45069858. Additionally, previously published metabolic data that were analyzed during this study are available in reference 8 and its supplemental information file.

## SUPPLEMENTAL MATERIAL

Supplemental figures msystems.00843-25-s0001.pdf Figures S1 to S4.

Table S1 msystems.00843-25-s0002.xlsx Species-level FPKM values from phyllosphere transcriptomes.

Table S2 msystems.00843-25-s0003.xlsx Effects of rust developmental stages on bacterial, fungal, and viral community alpha-diversity of diseased and healthy leaves.

Table S3 msystems.00843-25-s0004.xlsx Effects of leaf condition and sampling stage on the structure of viral transcriptomes.

Table S4 msystems.00843-25-s0005.xlsx DEGs at healthy and diseased stages.

Table S5 msystems.00843-25-s0006.xlsx Functional annotation of transcripts based on the KEGG database.

Table S6 msystems.00843-25-s0007.xlsx Functional annotation of transcripts based on the GO database.

Table S7 msystems.00843-25-s0008.xlsx Functional annotation of transcripts based on the CAZy database.

Table S8 msystems.00843-25-s0009.xlsx Importance of phyllosphere microbial functional genes in the severity of crabapple rust disease determined in random forest analysis.

Table S9 msystems.00843-25-s0010.xlsx Metatranscriptomic sequencing and bioinformatics analysis results from 36 RNA libraries.

ASM does not own the copyrights to Supplemental Material that may be linked to, or accessed through, an article. The authors have granted ASM a non-exclusive, world-wide license to publish the Supplemental Material files. Please contact the corresponding author directly for reuse.
