# Supplementary material for: Functional dynamics reveal the response of the crabapple (Malus sp.) phyllosphere microbiome to Gymnosporangium yamadae infection
Source: mSystems. 2025 Sep 15;10(10):e00843-25. doi: 10.1128/msystems.00843-25 (PMC12542627; doi:10.1128/msystems.00843-25)
Supplement: Supplemental figures — Figures S1 to S4. [file msystems.00843-25-s0001.pdf]

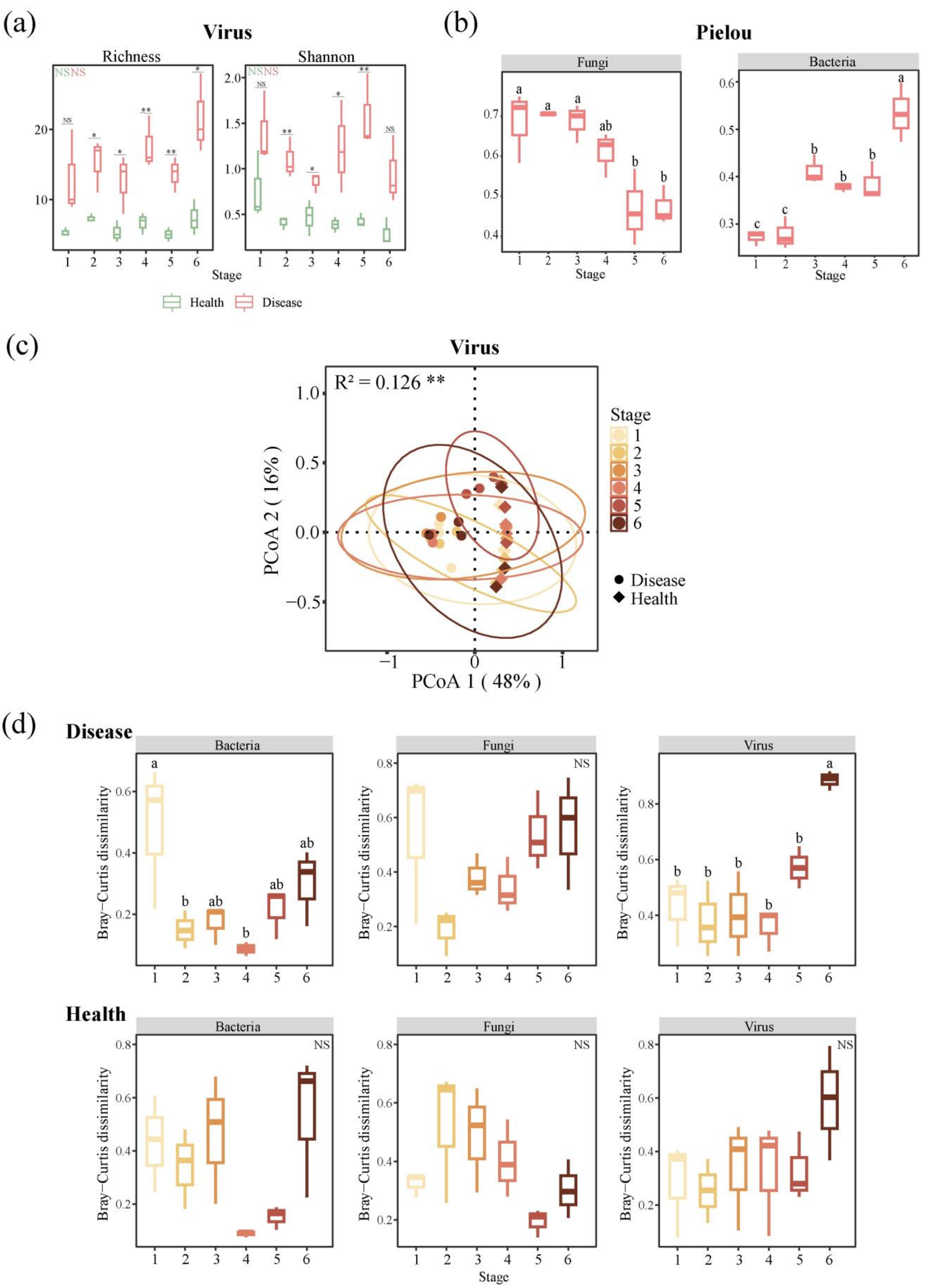
(a)
Virus
Richness
Shannon
Stage
Health
Disease
(b)
Pielou
Fungi
Bacteria
Stage
(c)
Virus
R² = 0.126 **
PCoA 2 (16%)
PCoA 1 (48%)
Stage
Disease
Health
(d)
Disease
Bacteria
Fungi
Virus
Bray−Curtis dissimilarity
Health
Stage

(e)

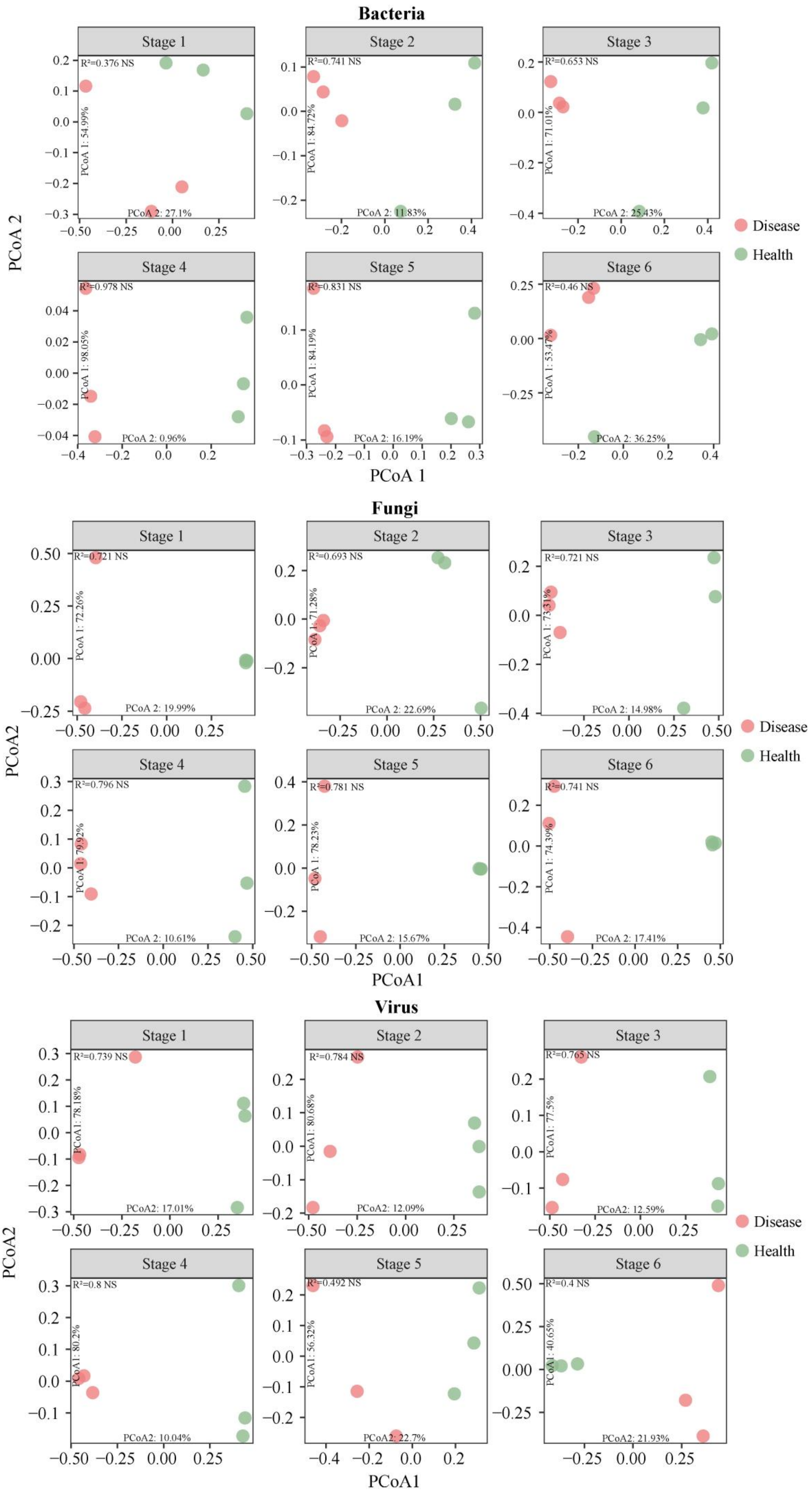

Bacteria
Stage 1
R²=0.376 NS
PCoA 1: 54.99%
PCoA 2: 27.1%
Stage 2
R²=0.741 NS
PCoA 1: 84.72%
PCoA 2: 11.83%
Stage 3
R²=0.653 NS
PCoA 1: 71.01%
PCoA 2: 25.43%
Stage 4
R²=0.978 NS
PCoA 1: 98.05%
PCoA 2: 0.96%
Stage 5
R²=0.831 NS
PCoA 1: 84.19%
PCoA 2: 16.19%
Stage 6
R²=0.46 NS
PCoA 1: 53.47%
PCoA 2: 36.25%
PCoA 2
PCoA 1
Disease
Health
Fungi
Stage 1
R²=0.721 NS
PCoA 1: 72.26%
PCoA 2: 19.99%
Stage 2
R²=0.693 NS
PCoA 1: 71.28%
PCoA 2: 22.69%
Stage 3
R²=0.721 NS
PCoA 1: 73.31%
PCoA 2: 14.98%
Stage 4
R²=0.796 NS
PCoA 1: 79.92%
PCoA 2: 10.61%
Stage 5
R²=0.781 NS
PCoA 1: 78.23%
PCoA 2: 15.67%
Stage 6
R²=0.741 NS
PCoA 1: 74.39%
PCoA 2: 17.41%
PCoA2
PCoA1
Disease
Health
Virus
Stage 1
R²=0.739 NS
PCoA1: 78.18%
PCoA2: 17.01%
Stage 2
R²=0.784 NS
PCoA1: 80.68%
PCoA2: 12.09%
Stage 3
R²=0.765 NS
PCoA1: 77.5%
PCoA2: 12.59%
Stage 4
R²=0.8 NS
PCoA1: 80.2%
PCoA2: 10.04%
Stage 5
R²=0.492 NS
PCoA1: 56.32%
PCoA2: 22.7%
Stage 6
R²=0.4 NS
PCoA1: 40.65%
PCoA2: 21.93%
PCoA2
PCoA1
Disease
Health

Figure S1 Phyllosphere transcriptomes alpha diversity and structural changes of bacteria, fungi and virus under *G. yamadae* invasion. (a) The alpha diversity of viral transcriptomes in diseased and healthy leaves across six developmental stages of crabapple rust disease. Box plots display the range of values between the 25th and 75th percentiles, along with the median, minimum, and maximum observed values within each data set. Different letters indicate statistically significant differences determined using one-way ANOVA with Tukey-HSD post hoc test ($P < 0.05$). (b) The alpha diversity of bacterial and fungal transcriptomes in diseased and healthy leaves at different disease stages. Box plots show the same diversity metrics as described above. (c) Principal Coordinates Analysis (PCoA) of viral transcriptomes based on the Bray-Curtis distance matrix, R^2 and P were calculated using PERMANOVA test. (d) Bray-Curtis dissimilarity of bacterial, fungal and viral transcriptomes in diseased leaves and healthy leaves across six developmental stages of crabapple rust disease. Box plots display the diversity metrics, with statistically significant differences indicated by different letters (one-way ANOVA with Tukey-HSD post hoc test, $P < 0.05$). The asterisks indicate P significance level, *$P < 0.05$, **$P < 0.01$, ***$P < 0.001$ and ****$P < 0.0001$ and NS denotes no statistical significance. (e) Principal Coordinates Analysis (PCoA) of transcriptomes based on the Bray-Curtis distance matrix, R^2 and P were calculated using PERMANOVA test. NS denotes no statistical significance.

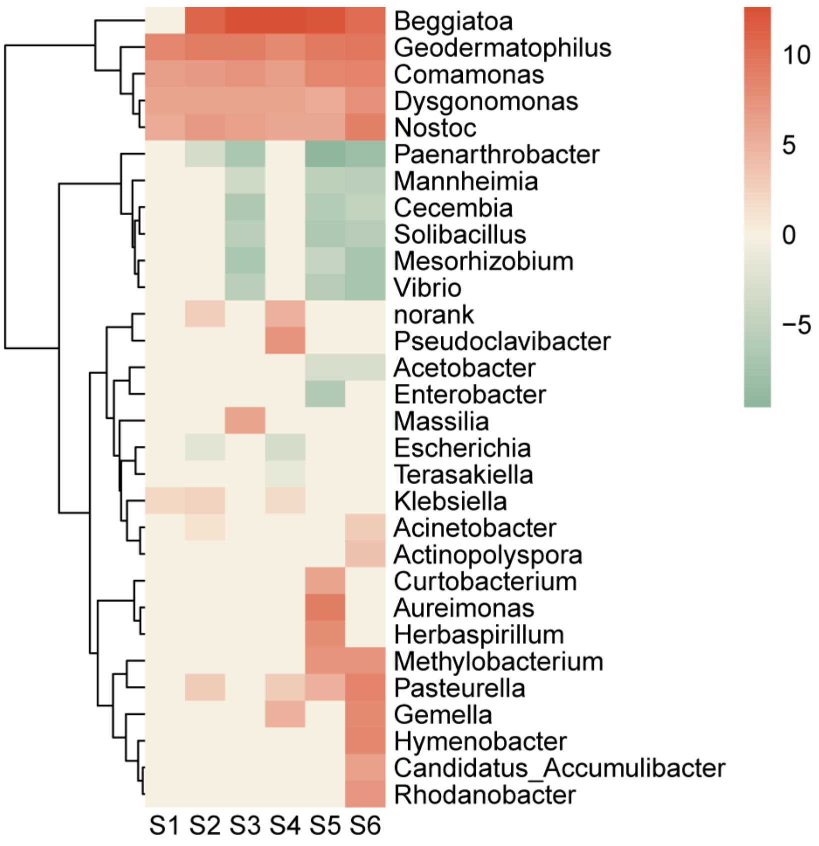

Figure S2 Significantly differentially expressed bacterial transcripts (based on genus level) in crabapple leaves with different conditions. Differential expression analysis based on generalized linear model (GLM) was used to identity transcripts showing significant differences at each developmental stage (*P* < 0.05, FDR corrected).

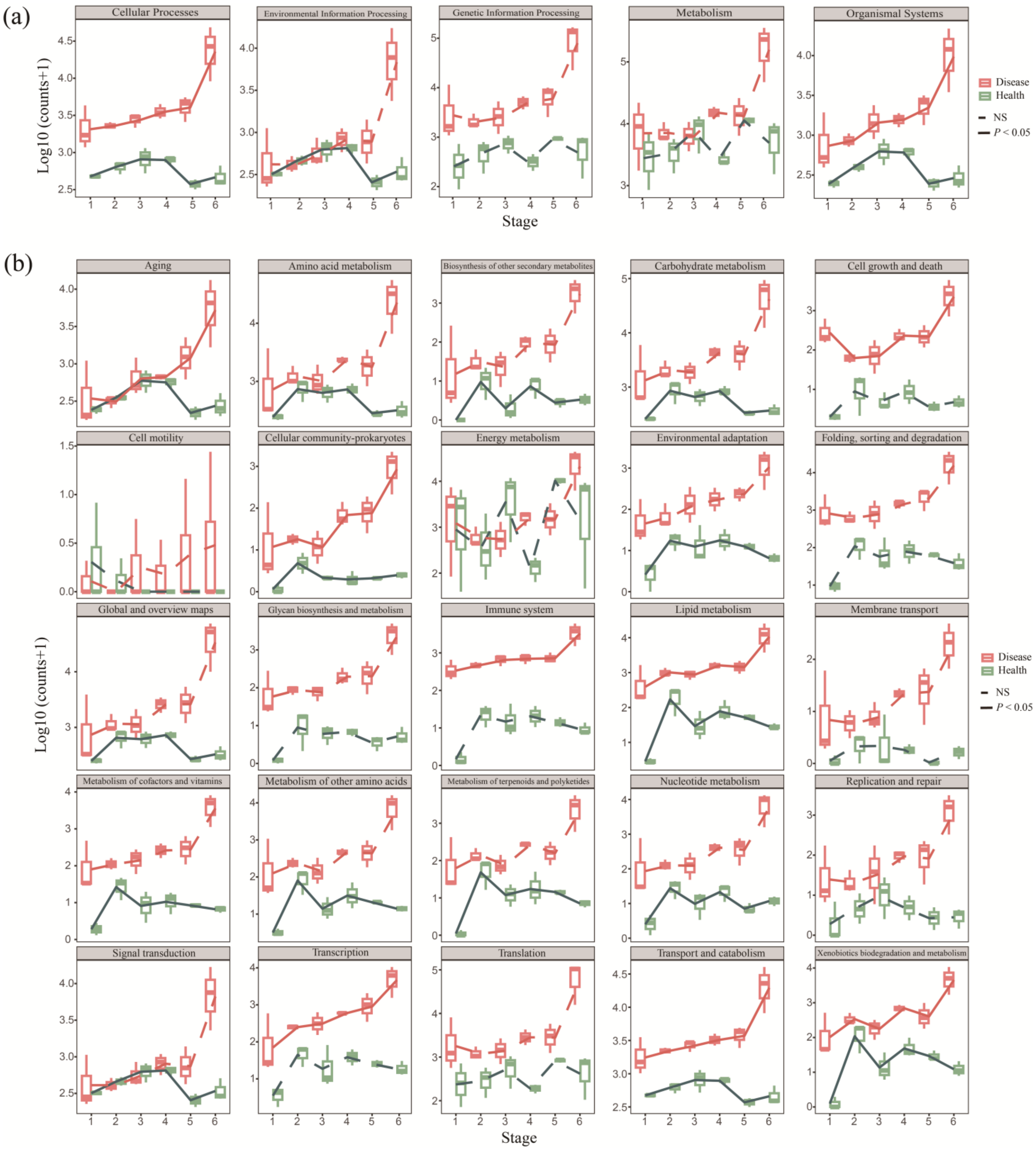

Figure S3 Dynamic expression profiles of functional genes in apple leaves with different conditions at KEGG (a) class 1 (b) and class 2 levels. Box plots show the range of estimated values between the 25th and 75th percentiles, with the median, minimum, and maximum observed values within each data set. The trend lines represent the mean values; solid lines indicate significant differences based on one-way ANOVA test ($P < 0.05$), while dashed lines indicate no significant differences ($P \geq 0.05$).

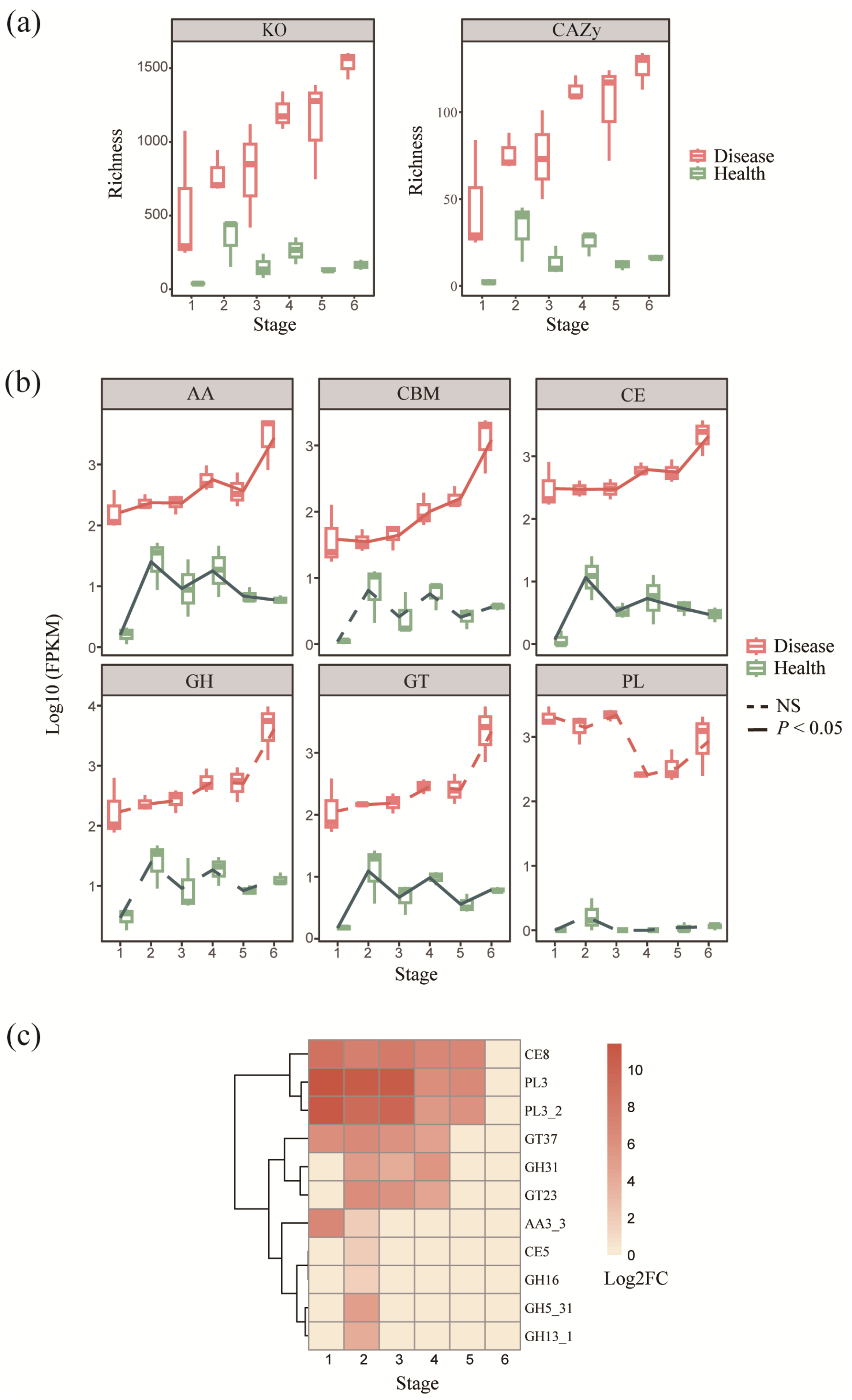

Figure S4 (a) Functional diversity between healthy and diseased phyllosphere microbiomes; diversity was characterized by richness indices focusing on KEGG Orthologs (KO) and CAZy. Box plots show the range of estimated values between the 25th and 75th percentiles, with the median, minimum, and maximum observed values within each data set. (b) Dynamic

expression profiles of functional genes in apple leaves with different conditions within each CAZy families. Trend lines represent the mean values; solid lines indicate significant differences based on one-way ANOVA test ($P < 0.05$), while dashed lines indicate no significant differences ($P \geq 0.05$). Box plots show the same diversity metrics as described above. (c) Significantly differentially expressed CAZymes at different stages of rust disease. Differential expression analysis based on generalized linear model (GLM) was used to identify transcripts showing significant differences at each developmental stage ($P < 0.05$, FDR corrected).
